# Corrosion Evaluation and Mechanism Research of AISI 8630 Steel in Offshore Oil and Gas Environments

**DOI:** 10.3390/ma17194907

**Published:** 2024-10-07

**Authors:** Zhao Zhang, Liang Wen, Que Huang, Li Guo, Zhizhong Dong, Lin Zhu

**Affiliations:** 1 School of Energy and Power Engineering, North University of China, Taiyuan 030051, China; zhangz_123@163.com; 2 Shanxi Key Laboratory of Efficient Hydrogen Storage & Production Technology and Application, School of Materials Science and Engineering, North University of China, Taiyuan 030051, China; 3 CRRC GUIYANG CO., Ltd., Guiyang 550017, China; wenliang6731@126.com; 4 School of Environment and Safety Engineering, North University of China, Taiyuan 030051, China; 5 School of Materials Science and Engineering, Tianjin University of Technology, Tianjin 300384, China; 6 Tianjin Heavy Equipment Engineering Co., Ltd., China First Heavy Industries Co., Ltd., Tianjin 300457, China

**Keywords:** composition optimization, corrosion resistance test, corrosion mechanism, AISI 8630 steel, marine corrosion and protection

## Abstract

In this study, we optimized the traditional composition of AISI 8630 steel and evaluated its corrosion resistance through a series of tests. We conducted corrosion tests in a 3.5% NaCl solution and performed a 720 h fixed-load tensile test in accordance with the NACE TM-0177-2016 standard to assess sulfide stress corrosion cracking (SSCC). To analyze the corrosion products and the structure of the corrosion film, we employed X-ray diffraction and transmission electron microscopy. The corrosion rate, characteristics of the corrosion products, structure of the corrosion film, and corrosion resistance mechanism of the material were investigated. The results indicate that the optimized AISI 8630 material demonstrates excellent corrosion resistance. After 720 h of exposure, the primary corrosion products were identified as chromium oxide, copper sulfide, iron oxide, and iron–nickel sulfide. The corrosion film exhibited a three-layer structure: the innermost layer with a thickness of 200–300 nm contained higher concentrations of alloying elements and formed a dense, cohesive rust layer that hindered the diffusion of oxygen and chloride ions, thus enhancing corrosion resistance. The middle layer was thicker and less rich in alloying elements, while the outer layer, approximately 300–400 nm thick, was relatively loose.

## 1. Introduction

Since the early 21st century, new oil and gas reserves have predominantly been discovered offshore, making offshore petroleum engineering a focal point of international competition [1]. The demand for energy resources has driven exploration into deeper and more hostile marine environments, where complex environmental conditions pose significant challenges to the durability and reliability of infrastructure. Offshore oil equipment, such as high-pressure wellhead units, must meet stringent performance criteria to withstand not only complex stress conditions but also aggressive corrosive agents like CO_2_, H_2_S, and chloride ions [2]. This creates a dual demand for materials that exhibit both mechanical strength and superior corrosion resistance, especially in high-stress areas like wellhead units and pipelines.

Marine corrosion is a persistent problem that affects the integrity of offshore structures. It is estimated that corrosion accounts for approximately 20% of total maintenance costs in offshore operations [3]. Moreover, the failure of critical components, such as wellheads and pipelines, due to corrosion can lead to catastrophic oil spills, environmental damage, and significant economic loss. The wellhead, as a central component in oil extraction, is subjected to some of the harshest conditions, including exposure to sour gasses (H_2_S), high chloride concentrations, and fluctuating temperatures and pressures. Therefore, selecting and optimizing materials for such applications is of paramount importance.

AISI 8630 quenched and tempered steel, widely used in high-pressure wellhead equipment, is part of the Cr-Mo steel family and is known for its low-alloy corrosion resistance. However, its low-alloy composition makes it cost-effective for many applications, though, like many materials used in marine environments, it is not immune to stress corrosion cracking (SCC), particularly in sour gas environments rich in hydrogen sulfide (H_2_S). The presence of H_2_S, especially in wet conditions, accelerates sulfide stress corrosion cracking (SSCC), which is a phenomenon that can drastically reduce the lifespan of critical components if not properly mitigated [4]. SSCC occurs when tensile stress and a corrosive environment combine to promote crack formation and growth. In wet H_2_S environments, H_2_S dissociates into H^+^ and HS^-^, with hydrogen atoms diffusing into the steel, leading to hydrogen embrittlement and subsequent cracking [5]. However, traditional AISI 8630 steel may exhibit only limited resistance to SSCC due to its relatively low alloy content and inability to form robust protective films in aggressive environments.

Performing corrosion resistance tests on wellhead equipment material is of great significance for the application of these materials in offshore oil and gas development. Existing studies indicate that higher material hardness [3] and specific alloying elements [6] are important factors affecting the SSCC sensitivity of wellhead equipment materials. Alloying elements like Mo, Cr, Ti, Nb, and V contribute to resistance by acting as hydrogen traps and enhancing material stability. In wet H_2_S environments, hydrogen sulfide dissociates into H^+^ and HS^−^, which can lead to hydrogen-induced cracking by enriching hydrogen at grain boundaries and defects. Zhang et al. [5] reported that S^2−^, produced by H_2_S ionization, formed a corrosion film with Fe^2+^ on the metal surface in an H_2_S environment, which hindered the repair of the passivation film.

The debate continues regarding the mechanisms of stress corrosion cracking in H_2_S environments. Murayama et al. [7] studied corrosion films and suggested that corrosion-resistant alloys forming specific corrosion product films—such as a Ni-S (outer layer) and Cr-O (inner layer)—show improved resistance. The role of alloying elements in the corrosion film structure significantly impacts resistance to hydrogen sulfide stress corrosion cracking [8]. Similarly, thermal treatments that promote the formation of fine-grained microstructures can enhance toughness and reduce crack propagation under stress. Researchers have explored various heat treatments, surface modifications, and alloying strategies to enhance the performance of materials like AISI 8630 steel. For instance, dual-phase steel, where austenite and ferrite are present, has been shown to offer improved corrosion resistance [9].

Despite these advancements, the complete understanding of these mechanisms and corrosion film characteristics under combined marine and wet H_2_S stress conditions remains incomplete. While increasing the hardness of steel may improve its mechanical strength, excessive hardness can increase susceptibility to SSCC. Therefore, an optimal balance must be achieved through the precise control of composition and processing conditions. Additionally, further research is needed to fully understand the long-term performance of optimized steel in real-world offshore environments.

In this paper, the AISI 8630 steel composition for deep-sea wellhead applications was optimized, and its corrosion resistance in a marine environment was examined through full immersion testing and SSCC performance evaluation. This study provides valuable insights into the corrosion film structure, characteristics, and mechanisms, offering a theoretical and experimental foundation for the development and application of corrosion-resistant steel in offshore oil and gas fields.

## 2. Experimental Method

The experimental materials used in this study are summarized in Table 1. To enhance the low-temperature toughness of AISI 8630 steel in marine environments, approximately 0.5% nickel was added to the traditional composition. Additionally, less than 0.05% vanadium was included to improve strength, toughness, and resistance to hydrogen sulfide stress corrosion. The carbon content was adjusted within medium and lower limit ranges to enhance material toughness, while the chromium and molybdenum contents were set at the upper limit to preserve hardening ability and strength while improving corrosion resistance. The sulfur and phosphorus contents were strictly controlled to optimize overall material performance.

The material was melted and cast in a vacuum induction furnace in a laboratory setting. After homogenization at 1200 °C, it was forged and subjected to heat treatment. The performance of the final material was assessed, yielding the following properties: tensile strength (*σb*) of 893 MPa, yield strength (*σs*) of 825 MPa, elongation (*δ*) of 20.5%, reduction in the area (*Ψ*) of 73%, impact toughness (Akv) of 207 J at −18 °C, and hardness (HRC) of 23.5.

### 2.1. Corrosion Rate Measurement

For the corrosion rate measurement, samples with dimensions of 10 mm × 10 mm × 10 mm were used. Three samples with the same composition were selected at each time point to verify the reliability of the results. These samples were fully immersed and suspended in a 3.5% NaCl solution at room temperature for 180 days. Prior to immersion, the samples were sanded to 1000 mesh, washed with distilled water, dried with alcohol, and weighed, recording the average weight as *M*_0_. After immersion, the samples were cleaned with anhydrous ethanol, dried, and weighed again, denoted as *M*_1_. The weight loss (ΔW) was calculated as *M*_0_–*M*_1_, and the corrosion rate was determined using the following equation:

(1) $$R=\frac{K\times (M_{0}-M_{1})}{S\times T\times D}$$

where *R*—the corrosion rate, mm/year;
*M*_0_—the mass of the sample before the test, g;
*M*_1_—the mass of the sample after the test, g;
*S*—the total area of the sample, cm^2^;
*T*—the test time, h;
*D*—the density of the material, kg/m^3^;
*K*—a constant equal to 8.76 × 10^7^.

### 2.2. Hydrogen Sulfide Stress Corrosion Cracking Test

Hydrogen sulfide stress corrosion cracking (SSCC) resistance was evaluated according to the NACE TM-0177-2016 standard [10]. A fixed load tensile test was conducted in an SSCC area with a stress set at 90% of the yield point (RP0.2). The test solution comprised 5 wt% NaCl and 0.4% sodium acetate, with a pH of 4.5 adjusted using HCl or NaOH. The test setup was purged with nitrogen to remove oxygen, followed by hydrogen sulfide to achieve a partial pressure of 0.01 MPa. The test was conducted at 24 ± 3 °C, with three samples assessed for SSCC over a period of 720 h.

### 2.3. Macroscopic Observation and X-ray Diffraction

Following the SSCC test, corrosion products were removed from the sample surfaces. The samples were then observed under an AXIOVERT 200MAT optical microscope for macroscopic examination. The corrosion products were mounted on a glass slide, and phase analysis of the corrosion film was performed using an X’Pert Pro X-ray diffractometer (Panalytical B.V, Max-Planck-Institute, Munich, Germany).

### 2.4. Sample Preparation and Transmission Electron Microscopy

**Figure 1:**
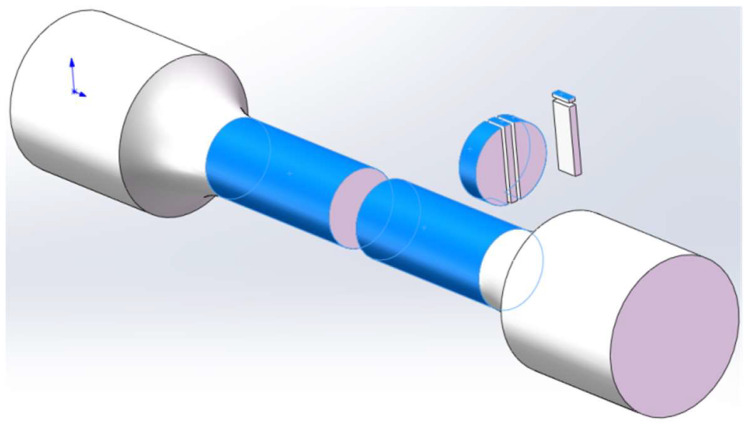
Schematic diagram of the FIB preparation system used for corrosion samples.

For transmission electron microscopy (TEM) analysis, samples were prepared using an FEI FIB200 focused ion beam system. A long rod-shaped sample, approximately 10 mm in length, was cut from the corrosion-exposed sample. This rod was then sliced longitudinally into 2 mm thick sections. A 1–2 µm thick platinum film was deposited on the surface of the corrosion layer to protect it during thinning. Initial thinning was performed using Ga ion sputtering. When the sample thickness reached 2–5 µm, the ion beam current was reduced from 6 nA to 3 nA, achieving a final thickness of approximately 3 µm. The samples were analyzed for diffraction and energy spectroscopy using a JEM-2100 field emission transmission electron microscope (JEOL, Tokyo, Japan). The sample preparation process is illustrated in Figure 1.

## 3. The Results

### 3.1. Corrosion Rate Analysis

**Figure 2:**
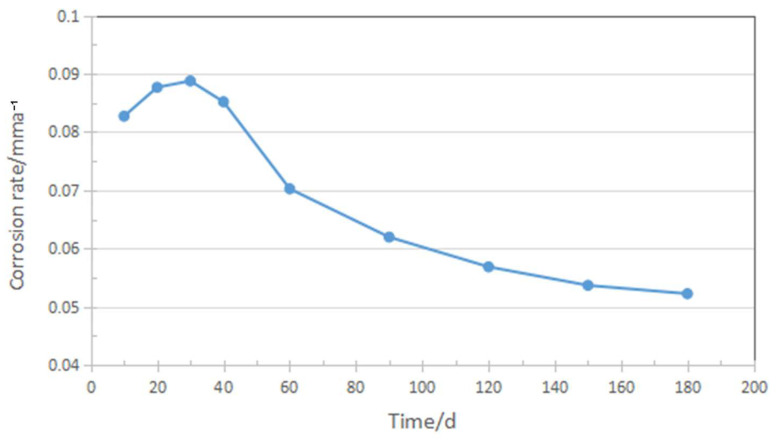
Corrosion rates of the improved AISI 8630 steel.

Figure 2 illustrates the corrosion rate of enhanced AISI 8630 steel, which was immersed in a 3.5% NaCl solution for 180 days following normalization, quenching, and tempering heat treatments. Initially, the corrosion rate increased, peaking at around 30 days. Subsequently, the rate decreased from approximately 0.09 mm/year at 30 days to about 0.05 mm/year by 180 days, eventually stabilizing. It is reported that [10] unoptimized AISI 8630 steel exposed to a 3.5% NaCl solution shows a corrosion rate of approximately 0.18 mm/year after 720 h of immersion. The corrosion products were primarily composed of iron oxides, and the corrosion film that formed was relatively loose and porous, leading to insufficient protection against chloride ion penetration. The improvement in corrosion behavior in this study is attributed to the development of a thicker and more adherent corrosion film over time. Initially, the film was thin and relatively loose, but as corrosion progressed, the accumulation of corrosion products thickened the film and improved its adhesion to steel [11]. This enhanced protective layer reduces the corrosion rate, indicating the improved resistance of AISI 8630 steel in marine environments.

### 3.2. Macroscopic Examination after Corrosion Testing

**Figure 3:**
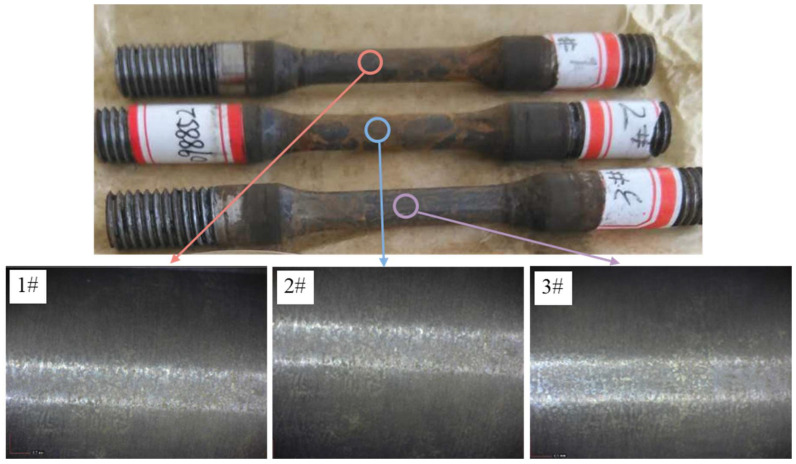
Macroscopic morphology of the studied samples after 720 h of the SSCC test (1#, 2# and 3# samples were obtained from the same parallel experiment).

The macroscopic examination of samples after 720 h of sulfide stress corrosion cracking (SSCC) test is shown in Figure 3. It is indicated that the samples did not exhibit fractures, and the surface was covered with a dark-brown corrosion product film. This film was relatively loose, and some of it detached, suggesting the poor adhesion and limited protective capability of the corrosion layer. The magnified macroscopic examination of the samples after removal of the corrosion products is shown at the bottom of Figure 3. No cracks were observed on the surface of any of the three samples, meeting the NACE TM-0177-2016 evaluation standard for SSCC. Thus, improved AISI 8630 steel demonstrated excellent resistance to hydrogen sulfide stress corrosion cracking.

### 3.3. X-ray Diffraction Analysis of Corrosion Products

**Figure 4:**
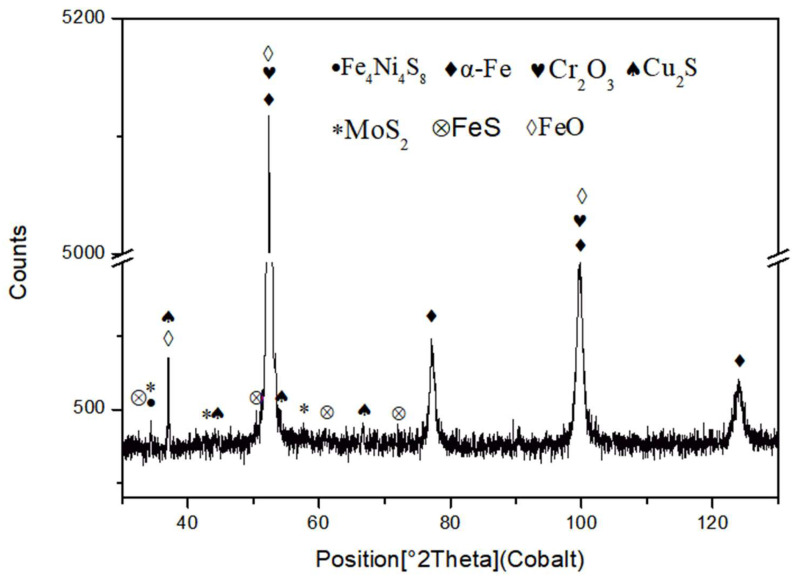
XRD patterns of the corrosion product film on the sample surface after the SSCC test.

X-ray diffraction (XRD) analysis was performed on the corrosion products of the improved AISI 8630 steel after SSCC testing, as shown in Figure 4. The XRD pattern revealed the presence of various phases, including α-Fe, FeS, Cr_2_O_3_, Cu_2_S, MoS_2_, Fe_4_Ni_4_S_8_, and FeO. The diffraction peaks for α-Fe, FeO, and Cr_2_O_3_ were prominent, indicating their substantial presence in the corrosion products. Conversely, FeS, MoS_2_, Cu_2_S, and Fe_4_Ni_4_S_8_ were present in lower quantities. FeS formed by the reaction between H_2_S and iron in a weakly acidic medium, creating an unstable and porous film with poor corrosion resistance [12]. In contrast, Cr_2_O_3_, chromium oxide, provided strong resistance to hydrogen sulfide, enhancing the corrosion resistance of the steel. The presence of Fe_4_Ni_4_S_8_ suggested that a compound formed from iron, nickel, and sulfur, with nickel contributing to the stability of the corrosion film due to its resistance to oxidation [13]. Similarly, molybdenum (Mo), which is also resistant to oxidation, contributed to the stability of the film by forming MoS_2_ [14]. The relatively low levels of MoS_2_ and Cu_2_S in the corrosion film corresponded to the lower concentrations of molybdenum and copper in the enhanced AISI 8630 steel.

### 3.4. Transmission Electron Microscopy of the Corrosion Film

**Figure 5:**
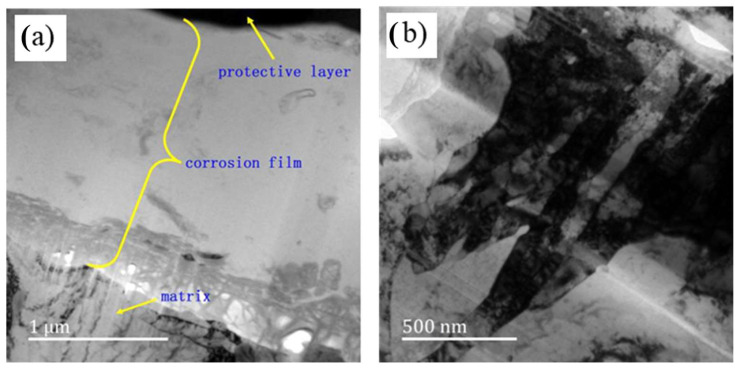
Transmission electron microscopy images showing the morphology of the matrix and corrosion layer: morphology of the protective corrosion film (**a**) and morphology of the matrix (**b**).

Figure 5 presents the morphology of the corrosion film on the sample surface after 720 h of H_2_S stress corrosion testing, as observed using transmission electron microscopy (TEM). The images reveal a distinct contrast between the corrosion film and the underlying matrix. The outer layer of the corrosion film, which appears as a black protective layer (top right corner), results from focused ion beam (FIB) cutting. The matrix is situated beneath this corrosion film. The film varies in thickness, approximately 2–3 µm, and is lighter in color compared to the matrix. The TEM images show a notable dark region between the surface and the inner layer near the matrix. The middle layer of the film exhibits low contrast, while the inner layer contains dispersed black corrosive particles. The matrix displays clear bainite laths, grain boundaries, and high-density dislocations attributed to stress.

**Figure 6:**
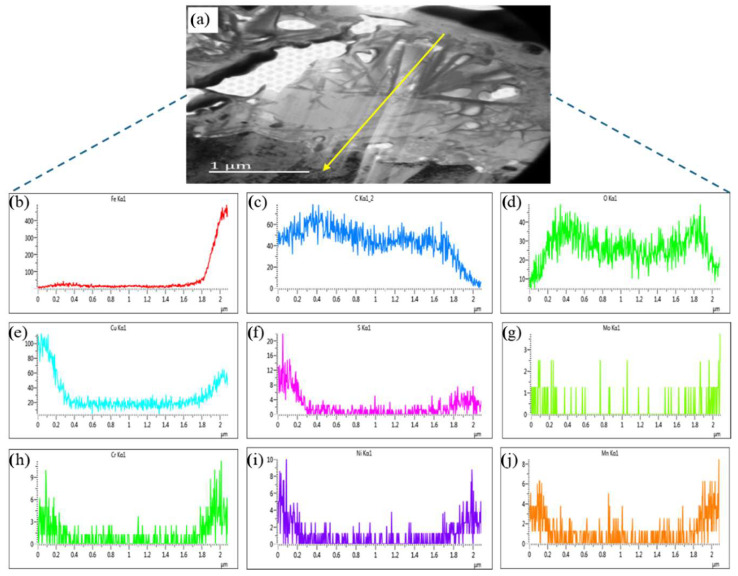
The morphology of the corrosion layer (**a**) and corresponding line scanning analyses (along the yellow line): Iron (**b**), Carbon (**c**), Oxygen (**d**), Copper (**e**), Sulfur (**f**), Molybdenum (**g**), Chromium (**h**), Nickel (**i**), and Manganese (**j**).

Line scanning analysis was conducted from the outer layer of the corrosion film to the matrix (along the yellow line illustrated in Figure 6a) to determine the distribution of elements. The elemental distribution results are displayed in Figure 6b–j, respectively. It is indicated that the distribution of α-Fe in the corrosion film varies, with a higher Fe content observed at 0–600 nm from the surface and at 1800–2000 nm near the matrix (Figure 6b). The Fe content is relatively lower in the middle of the film but still significant compared to other elements. The carbon (C) content is higher in the corrosion film than in the matrix (Figure 6c). Oxygen (O) exhibits a “W”-shaped distribution, with higher concentrations at 400 nm below the surface and at 1800 nm near the matrix but lower in the middle of the corrosion film (Figure 6d).

As seen in Figure 6e–g, copper (Cu), sulfur (S), and molybdenum (Mo) are concentrated in two main regions: the surface layer (0–300 nm) and the transition zone between the corrosion film and the matrix (1800–2000 nm). The iron (Fe) content peaks at the surface of the corrosion film and is lowest in the middle layer. Chromium (Cr) distribution mirrors that of Cu and S but with a peak observed at 1900 nm near the matrix (Figure 6h). A Cr-O film, identified as Cr_2_O_3_, forms in the inner layer of the corrosion film [15]. The distribution of manganese (Mn) and nickel (Ni) elements are displayed in Figure 6i,j and exhibit similar distribution patterns, with significant enrichment at the surface (0–300 nm) and slightly higher levels near the matrix (1800–2100 nm).

## 4. Analysis and Discussion

### 4.1. Analysis of the Immersion Corrosion Rate

The corrosion rate of the material is primarily influenced by the performance of the corrosion film formed on its surface. Initially, a corrosion film develops, primarily composed of iron oxide, which is loose and prone to peeling and offers some isolation from the corrosive environment. Tewary et al. [16] indicated that early-stage corrosion films are typically iron oxides, which have a limited protective effect due to their loose nature.

In marine environments, chromium (Cr) compounds offer protective benefits, but Cr-containing films are relatively thin in the initial stages of corrosion. Chloride ions (Cl^−^) in seawater can compromise the protective ability of these films, disrupting passivation and making the interface more active [17,18]. Consequently, the early-stage corrosion film does not effectively prevent corrosive media from reaching the material, leading to accelerated corrosion [19]. As corrosion progresses, the corrosion film thickens, particularly in its inner layer, improving its protective capability. This increased thickness of the corrosion layer reduces the exposure of the matrix to the corrosive solution and decreases the hydrogen atom’s permeability. Consequently, the corrosion rate decreases after an initial peak and stabilizes as the film reaches a more protective state [20].

### 4.2. Analysis of SSCC Products

**Figure 7:**
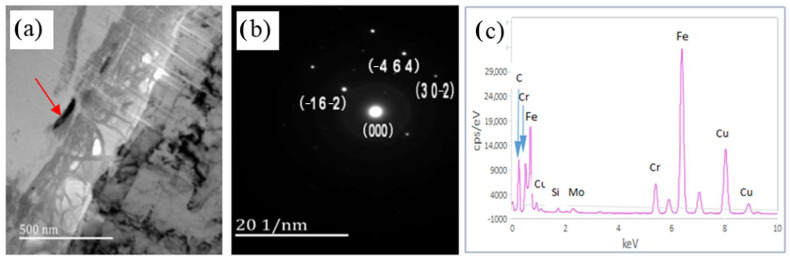
Morphology of Cr_2_O_3_ oxide particles in the corrosion film (**a**) and its corresponding diffraction pattern (**b**) and energy spectrum pattern (**c**).

The X-ray diffraction (XRD) results reveal that the primary corrosion products on the sample surface are sulfides, iron and chromium oxides, and iron–nickel compounds. The formation of these products is closely related to the type of steel, the composition of the solution, the pH value, and the temperature of the solution [21]. Some corrosion products, such as chromium compounds and iron–chromium compounds, offer protective benefits, while others, like iron oxide, are less effective. Although iron sulfide, formed from hydrogen sulfide (H_2_S) and iron, can act as a protective film under certain conditions, it is not very dense and does not strongly prevent stress corrosion cracking (SSCC) [6]. Figure 7 illustrates black corrosion particles within the inner layer of the corrosion film close to the matrix. The energy spectrum and diffraction analyses identify these particles as Cr_2_O_3_ with a hexagonal crystal structure. The presence of crystalline chromium oxide indicates chromium enrichment in the inner layer of the corrosion film, which contributes to a dense and stable protective layer. This layer effectively prevents hydrogen and iron ion reactions under the film, improving steel’s resistance to sulfuric, hydrochloric, and certain organic acids, as well as enhancing pitting resistance [22]. The line scanning analysis confirms chromium enrichment near the matrix, which is consistent with previous research findings [15]. While molybdenum sulfide (MoS_2_) is present in low quantities, molybdenum (Mo) is known for its strong carbide-forming properties, which help form a dense passive film on steel surfaces and impede the reaction between H_2_S and the material [23]. Therefore, chromium and molybdenum are crucial for improving hydrogen sulfide stress corrosion resistance. However, excessive amounts of these elements can lead to larger carbide particles, which can negatively affect steel’s resistance to SSCC [24].

Copper, primarily present in the form of Cu_2_S, also plays an important role despite its minimal quantity. Copper accelerates the recombination of hydrogen atoms, reducing their activity and promoting the formation of passive films [25]. Additionally, copper enhances corrosion and pitting resistance in acidic environments, which is beneficial for SSCC resistance [26]. It also supports the formation of Cr_2_O_3_, further preventing steel dissolution and corrosion. The XRD results also indicate the presence of Fe_4_Ni_4_S_8_, which is a sulfide of iron and nickel. Nickel (Ni) contributes to higher corrosion potential and lower corrosion current, forming a dense oxide film in acidic conditions. This film reduces hydrogen atom ingress into the steel matrix and slows H_2_S corrosion [27]. However, the low hydrogen evolution potential of nickel-containing steel can facilitate hydrogen ion reduction, leading to increased free hydrogen precipitation and compromising resistance to sulfide stress corrosion. For effective H_2_S corrosion resistance, nickel content should not exceed 1% [6]. Improved AISI 8630 steel, with a nickel content of 0.55%, meets this requirement.

### 4.3. Analysis of the Corrosion Film after SSCC

**Figure 8:**
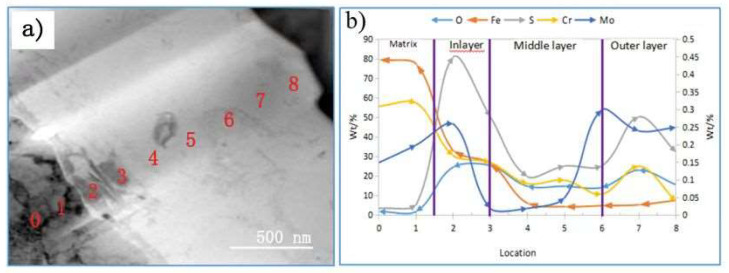
Morphology (**a**) and elemental distribution analysis (**b**) of the corrosion film.

To further examine the structure of the corrosion film and validate the line scanning results, energy spectrum analysis was conducted using transmission electron microscopy (TEM), as illustrated in Figure 8. The results indicate that the element distribution in the corrosion layer aligns closely with the line scanning data. The matrix displays a higher concentration of α-Fe compared to the corrosion film, indicating the predominant presence of α-Fe in the matrix. In the corrosion film, iron reacts with oxygen (O) and sulfur (S) to form FeO and FeS. Oxygen is primarily concentrated in both the inner and outer layers of the film, corroborating the presence of chromium oxide (Cr_2_O_3_) [28]. The distributions of Cr and O are similar, with both elements enriching the outer and inner layers, thus contributing to the formation of a protective Cr-O film that enhances steel’s corrosion resistance. This film structure resembles the three-layer model described by Masakatsu [29].

As shown in Figure 8b, molybdenum (Mo) is predominantly found in the inner and outer layers, with the highest concentration in the outer layer. Mo forms MoS_2_ with sulfur, which is consistent with the XRD findings. Mo stabilizes the Cr-O corrosion film and enhances the material’s strength while preventing phosphorus segregation, thereby improving overall corrosion resistance [30]. The energy spectrum analysis indicates that the sulfur content in the corrosion film is approximately 20 to 30 times higher than in the base metal, suggesting that the S in the corrosion film primarily originates from the hydrogen sulfide (H_2_S) solution. Sulfur compounds in the inner layer of the base metal likely form during the early stages of corrosion. As corrosion progresses, sulfur-containing particles gradually accumulate, resulting in the high sulfur content observed in the inner corrosion layer [31].

Line scanning results (shown in Figure 8b) reveal that carbon (C) is mainly distributed within the corrosion film, with a low concentration near the base metal. The carbon in the corrosion film may derive from the martensite–austenite (M-A) unit and C-rich austenite in the material. Due to the potential difference between the M-A unit and the matrix, along with the microgalvanic corrosion effect, the matrix around the M-A island undergoes preferential anodic dissolution early in the corrosion process, leaving behind the M-A unit and C-rich austenite region [32]. Nickel (Ni) is predominantly found in both the inner and outer layers of the corrosion film. Since the basic components of the M-A unit are carbon, manganese (Mn), and nickel, the Mn and Ni in the outer layer likely originate from the M-A unit [33]. Ni in the matrix forms Fe-Ni compounds with iron (Fe), contributing to the corrosion resistance of the inner layer. This multi-layered structure acts as a barrier, slowing down the diffusion of corrosive species into the steel substrate and reducing the likelihood of crack initiation. Such structures not only protect the steel from aggressive seawater but also improve its overall longevity and performance in harsh conditions.

**Figure 9:**
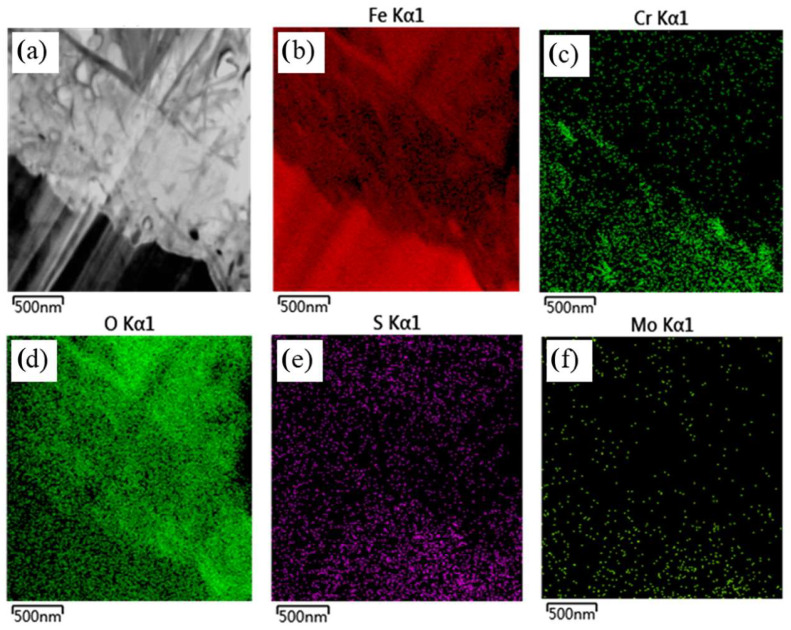
Elemental distribution of Fe, Cr, O, S, and Mo on the surface of corrosion film.

Surface scanning analysis was used to further examine the corrosion film structure, as shown in Figure 9. Unlike line scanning, surface scanning covers a larger area and provides a more intuitive overview. It is indicated that the α-Fe content is higher in the outer layer of the corrosion film and lower in the middle layer, excluding the matrix. This difference in iron content distribution between the outer and inner layers compared to the line scanning results may be due to the more comprehensive coverage of surface scanning. Additionally, the Cr content is higher in the inner layer of the corrosion film, forming a dense Cr-O film with strong corrosion resistance. The distribution of Fe, Cr, O, S, and Mo observed in surface scanning is consistent with line scanning and energy spectrum analysis, indicating that the elemental content is higher in the inner layer than in the outer layer, with the middle layer having the lowest content. Thus, the corrosion film has a three-layer structure.

Based on the distribution of Cr, Cu, Mo, Ni, Mn, and other elements, the corrosion film can be divided into three layers. The inner layer, closest to the base metal, has the highest elemental content and is relatively thin. The outer and middle layers are thicker, with the outer layer containing more elements than the middle layer.

## 5. Conclusions

1. Corrosion tests were conducted on improved AISI 8630 steel in a 3.5% NaCl solution. A 720-h fixed-load tensile test was performed to evaluate its susceptibility to sulfide stress corrosion cracking (SSCC), following the NACE TM-0177-2016 standard. The results showed a decrease in the corrosion rate from approximately 0.09 mm/year at 30 days to about 0.05 mm/year after 180 days. This reduction is attributed to the formation of a thicker and more adherent corrosion film over time.
2. After 720 h of the SSCC test, the corrosion products on the sample surface mainly consisted of FeO, FeS, Cr_2_O_3_, MoS_2_, and Cu_2_S. The corrosion film comprising iron oxides and sulfides were loose and porous, whereas the layers of Cr_2_O_3_ and Cu_2_S formed a dense, well-adhered rust layer. This dense film significantly contributes to improved corrosion resistance.
3. The corrosion film on the SSCC sample after 720 h exhibited a three-layer structure. The innermost layer, with a thickness of 200–300 nm, contained higher concentrations of alloying elements and formed a dense, cohesive rust layer that hindered the diffusion of oxygen and chloride ions, thus enhancing corrosion resistance. The middle layer was thicker and less rich in alloying elements, while the outer layer was loose and porous. This multi-layered structure acts as a barrier, slowing down the diffusion of corrosive species into the steel substrate and reducing crack initiation.

## Figures and Tables

**Table 1:** Chemical composition of the test material in wt%.

Element	C	Mn	Si	Cr	Mo	Ni	P	S	V	Cu
Actual	0.28	0.78	0.21	0.90	0.45	0.55	0.0048	0.0031	0.031	0.015

## Data Availability

The data presented in this study are available on request from the corresponding author due to (specify the reason for the restriction).
